# Does Well-Controlled Type 2 Diabetes Mellitus Affect Corneal Endothelium? A Comparative Study

**DOI:** 10.3390/jcm14155194

**Published:** 2025-07-22

**Authors:** Aleksandra Opala, Łukasz Kołodziejski, Iwona Grabska-Liberek

**Affiliations:** Department of Ophthalmology, Centre of Postgraduate Medical Education, 01-813 Warsaw, Poland

**Keywords:** type 2 diabetes mellitus, corneal endothelium, endothelial cell density, specular microscopy, central corneal thickness

## Abstract

**Background/Objectives:** The aim of this study is to compare the parameters of the corneal endothelium in a group of patients with well-controlled type 2 diabetes (study group) to those of patients who do not have type 2 diabetes (control group). **Methods:** The study aims to compare the corneal endothelium parameters of 80 eyes (80 patients) with well-controlled type 2 diabetes to 80 eyes (80 patients) without type 2 diabetes. The endothelial cell density (ECD), percentage of hexagonal cells (%HEX), coefficient of variation in cell size (CV), and central corneal thickness (CCT) were recorded using a non-contact specular microscope (Nidek CEM-530, Nidek Co., Ltd., Gamagori, Japan). Best-corrected visual acuity (BCVA) and intraocular pressure (IOP) were also measured. **Results:** The groups were matched for age and sex. A significantly lower ECD value was observed in the group of patients with type 2 diabetes (2480.76 cells/mm^2^ ± 303.48) compared to the control group (2629.64 cells/mm^2^ ± 304.73) (*p* = 0.002). BCVA was also significantly lower in the study group (0.44 ± 0.18) compared to the control group (0.50 ± 0.19) (*p* = 0.049). No statistically significant differences were found between the groups in terms of IOP, CV, %HEX, and CCT. **Conclusions:** Patients with well-controlled type 2 diabetes exhibit a lower ECD compared to individuals without diabetes, even in the absence of advanced diabetic complications. These subtle changes may have clinical implications for preoperative evaluation and long-term management in diabetic patients. The other morphological parameters of the corneal endothelium remain comparable between the groups.

## 1. Introduction

The global prevalence of diabetes continues to rise at an alarming rate. According to the International Diabetes Federation (IDF), an estimated 537 million adults (aged 20–79 years) were living with diabetes worldwide in 2021. This number is projected to increase to 643 million by 2030 and 783 million by 2045 [1]. The World Health Organization (WHO) reports that diabetes remains a leading cause of blindness, kidney failure, heart attacks, stroke, and lower limb amputation [2]. In developed countries, diabetes-related ocular complications are among the most common causes of visual impairment and blindness [3].

According to the American Diabetes Association (ADA), a glycated hemoglobin (HbA1c) level below 7% is considered an appropriate target for good glycemic control in most nonpregnant adults with type 2 diabetes. This threshold has been shown to reduce the risk of microvascular complications and, when implemented early, is associated with long-term macrovascular benefits.

Typical corneal changes associated with diabetes include the loss of long corneal nerves, thickening of the corneal stroma, and thinning of the corneal epithelium. As a result of these structural alterations, corneal sensitivity is often impaired, contributing to the development of punctate epithelial keratopathy, poorly healing epithelial defects, and recurrent erosions caused by disrupted epithelial regeneration [4,5,6].

Corneal endothelial cells are also affected by abnormal glycemic levels. Studies report a reduction in ECD and %HEX, accompanied by increased pleomorphism (variability in cell shape) and polymegethism (variability in cell size). These morphological changes may compromise the barrier and pump function of the endothelium, leading to corneal hydration imbalance and loss of transparency [7,8,9,10,11,12,13,14].

One proposed mechanism is the increased activity of aldose reductase under hyperglycemic conditions, resulting in the intracellular accumulation of sorbitol within epithelial and endothelial cells. This accumulation generates osmotic pressure, causing cellular swelling. Additional mechanisms of endothelial dysfunction include the build-up of advanced glycation end-products (AGEs), which induce oxidative stress and damage to cytoplasmic actin filaments [7,8]. Moreover, the slowdown of the Krebs cycle leads to reduced ATP production, impairing the active transport function of the endothelial pump system that maintains corneal stromal hydration [4,15]. Another factor is the increased expression of matrix metalloproteinases, which degrade the basement membrane and limit cell migration, further contributing to endothelial cell damage [5,16].

Given the variety of corneal alterations associated with diabetes mellitus and the proposed mechanisms of endothelial dysfunction, further investigation is needed to better understand the impact of glycemic control on the cornea, particularly in the absence of advanced diabetic complications. Therefore, the aim of this study was to assess the numerical and morphological parameters of corneal endothelial cells in patients with well-controlled type 2 diabetes compared to individuals without diabetes. In addition, the study aimed to evaluate CCT in both groups. By focusing on patients without diabetic retinopathy or with only mild nonproliferative changes, we sought to isolate the effects of stable glycemic disturbance on corneal structure and function.

## 2. Material and Methods

### 2.1. Patient Characteristics

The study participants were recruited from patients attending the Ophthalmology Clinic at the Centre of Postgraduate Medical Education in Warsaw and its affiliated outpatient clinic. The patients were divided into two groups:

1. Study group—patients diagnosed with well-controlled type 2 diabetes (HbA1c ≤ 7%).
2. Control group—patients without type 2 diabetes.

Each group consisted of 80 patients, making a total of 160 participants. For each patient, data from one eye was analyzed, resulting in a total of 160 eyes included in the study.

The study included patients aged 60–80 years. The diagnosis of type 2 diabetes was based on medical history and written confirmation from the attending physician. All diabetic patients were receiving oral antidiabetic medications during the study period. The medications used belonged to the following pharmacological groups: sulfonylureas, DPP-4 inhibitors, biguanides, thiazolidinediones, and SGLT2 inhibitors. The duration of type 2 diabetes in the study group ranged from 1 to 10 years. All participants were of Caucasian ethnicity.

In all patients with type 2 diabetes, glycated hemoglobin (HbA1c) levels were measured in serum to assess glycemic control.

In the group of patients with type 2 diabetes, no signs of diabetic retinopathy or only mild nonproliferative diabetic retinopathy (NPDR) were detected. None of the included patients had moderate or severe NPDR or proliferative diabetic retinopathy (PDR). The assessment and classification of diabetic retinopathy stages were conducted based on the Early Treatment of Diabetic Retinopathy Study (ETDRS) scale.

None of the patients had a history of previous retinal photocoagulation or treatment for diabetic macular edema (DME) with anti-VEGF agents.

Patients without diabetes were included in the study as the control group. Fasting blood glucose measurements were performed following the recommendations of the American Diabetes Association (ADA) to identify any undiagnosed cases of diabetes.

The inclusion and exclusion criteria are presented in Table 1.

### 2.2. Research Methods

This prospective study collected data on patient age and sex at the time of inclusion, along with a detailed medical history. At the time of inclusion in the study, the age and sex of each patient were recorded, and a medical history was taken, including information on the duration of type 2 diabetes, type of treatment, and the presence of comorbidities.All patients underwent a biomicroscopic examination to assess the anterior segment of the eye. The posterior segment and fundus were also evaluated based on the Early Treatment of Diabetic Retinopathy Study (ETDRS) classification.

The following measurements were conducted: BCVA using Snellen charts, IOP using Goldmann applanation tonometry (Haag-Streit AT 900 Goldmann Applanation Tonometer, Köniz, Switzerland), and ocular biometry using ultrasound biometry (Alcon OcuScan RxP Measuring System, software version 1.12).

ECD (cells/mm^2^) in the central corneal area, CV, %HEX, and CCT were analyzed using a non-contact specular microscope (Nidek CEM-530, Nidek Co., Ltd., Gamagori, Japan). During the measurement, three images of each cornea were taken. For further analysis, one image of satisfactory quality and with an average ECD value relative to the other two measurements was selected. All patients were of Caucasian ethnicity. For all patients with type 2 diabetes, glycated hemoglobin (HbA1c) levels were measured in serum to assess glycemic control. In the diabetic patient group, either no diabetic retinopathy was present or only mild nonproliferative diabetic retinopathy was observed. Non-diabetic patients were included in the study as the control group. Fasting blood glucose levels were measured in accordance with American Diabetes Association (ADA) guidelines to detect any previously undiagnosed diabetes. All patients included in the study had age-related cataracts at a stage qualifying for surgical treatment (nuclear density < grade 5 on the LOCS III scale), assessed using the Lens Opacities Classification System III (LOCS III).

The study protocol was approved by the Bioethics Committee of the Centre of Postgraduate Medical Education in Warsaw on 15 June 2020 (approval number 97/PB/2020). Informed consent for participation in the study was obtained from all patients included in the project.

### 2.3. Statistical Analysis

Statistical analysis was performed using R software (version 4.1.2). Qualitative data (gender) were described using absolute and relative frequencies, while quantitative data were presented as mean and standard deviation, median and interquartile range, as well as minimum and maximum values. The normality of distribution was assessed using the Shapiro–Wilk test, and if necessary, also by evaluating skewness and kurtosis coefficients. Homogeneity of variances was tested using Levene’s test. To compare groups, the following tests were applied: Student’s *t*-test (for normally distributed data with homogeneous variances), Mann–Whitney U test (when normality was not met), and Pearson’s chi-square test (for gender comparison). Differences were reported as mean or median differences with 95% confidence intervals. A significance level of α = 0.05 was adopted.

## 3. Results

### 3.1. Characteristics of the Analyzed Patient Groups

#### 3.1.1. Characteristics of the Group of Patients with Type 2 Diabetes (DM+)–Study Group

The study group consisted of 80 patients diagnosed with type 2 diabetes (DM+), of whom 53.8% were women. The mean age was 72.04 ± 5.60 years. The average HbA1c level was 6.77 ± 0.13%, confirming good glycemic control (HbA1c ≤ 7%). The mean IOP was 15.59 ± 2.84 mmHg, and the average BCVA was 0.44 ± 0.18.

The analysis included corneal endothelial parameters: the mean ECD was 2480.76 ± 303.48 cells/mm^2^, CV was 30.00 ± 4.81, %HEX was 68.21 ± 4.93%, and the mean CCT was 542.22 ± 27.94 μm. Detailed data are presented in Table 2.

#### 3.1.2. Characteristics of the Group of Patients Without Type 2 Diabetes (DM−)–Control Group

The control group consisted of 80 patients without type 2 diabetes (DM−), of whom 52.5% were women. The mean age was 73.22 ± 4.90 years. The average blood glucose level was 92.90 ± 4.08 mg/dL. The mean IOP was 15.38 ± 3.07 mmHg, and the average BCVA was 0.50 ± 0.19.

Corneal endothelial parameters were also analyzed: the mean ECD was 2629.64 ± 304.73 cells/mm^2^, CV was 29.12 ± 4.14, %HEX was 68.86 ± 4.59%, and the mean CCT was 548.20 ± 24.84 μm. Detailed data are presented in Table 3.

### 3.2. Comparison of Groups

**Figure 1:**
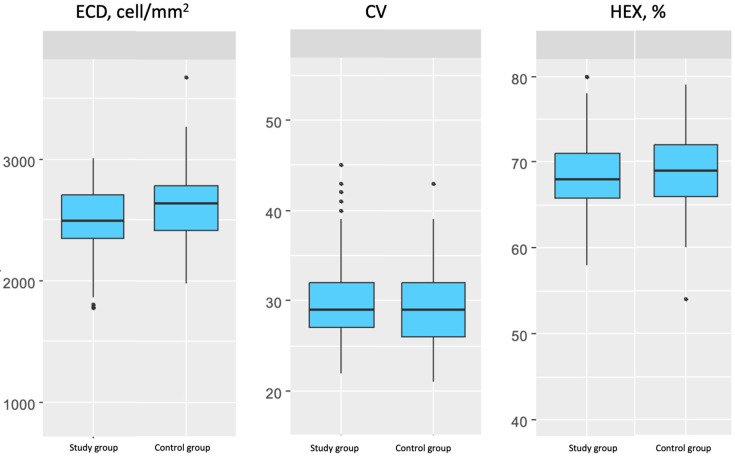
The distributions of endothelial cell density, coefficient of variation, and percentage of hexagonal cells in the study and control groups.

The comparison of groups showed no statistically significant differences in gender and age (*p* > 0.999 and *p* = 0.155, respectively). A statistically significant lower value was observed in the diabetic group for BCVA (mean difference = −0.06; 95% CI [−0.11; 0.00]; *p* = 0.049), with the *p*-value being at the threshold of statistical significance. ECD was also significantly lower in the diabetic group by 148.88 cells/mm^2^ (*p* = 0.002). Other parameters, including IOP and CCT, did not differ significantly between the groups (*p* > 0.05). A detailed comparison is presented in Table 4 and Figure 1.

## 4. Discussion

An analysis of the literature regarding the impact of diabetes on corneal endothelial cells reveals numerous discrepancies in the results obtained. Differences in findings across various studies may be attributed to ethnic background, type of diabetes (whether type 1 and type 2 were analyzed separately or together), duration of diabetes, and degree of glycemic control.

A significant portion of the studies conducted so far are retrospective and involve small patient groups. In some cases, patient selection criteria have been a point of controversy—many studies included both type 1 and type 2 diabetes patients, without considering the duration of diabetes, type of treatment, or effectiveness of therapy.

After analyzing the available literature, it was decided to design a study that addresses the limitations observed in previous research. The careful selection of inclusion and exclusion criteria aimed to achieve the most homogeneous patient population possible within the study.

This study was designed to include Caucasian patients within a specific age range. Only patients with type 2 diabetes, lasting between 1 and 10 years, with good glycemic control were intentionally included in the study.

Changes in the morphology and number of corneal endothelial cells, described using parameters such as ECD, CV, and %HEX, impact the endothelium’s ability to function properly as a protective barrier for the corneal stroma.

Abnormal endothelial cell morphology, combined with increased CCT, serves as a marker of endothelial dysfunction, which leads to fluid imbalance, corneal stroma edema, and loss of corneal transparency, ultimately limiting visual function.

### 4.1. Changes in Corneal Parameters in the Group of Patients with Type 2 Diabetes

The present study reports the status of corneal endothelial cells in a group of patients with type 2 diabetes, compared to a group of patients without diabetes.

### 4.2. Best-Corrected Visual Acuity (BCVA)

In this study, BCVA was recorded using Snellen charts. BCVA in the group of patients with type 2 diabetes was significantly lower than in the control group.

This difference may be attributed to a higher degree of lens opacification in the diabetic group or the location of the opacity. The comparison of BCVA between groups was valuable due to the relatively low probability of other causes of reduced BCVA in both groups.

It is important to emphasize that in the group of patients with type 2 diabetes, no diabetic retinopathy was detected, or only mild nonproliferative diabetic retinopathy (NPDR) was observed. None of the patients included in the study had moderate or severe NPDR or proliferative diabetic retinopathy (PDR).

### 4.3. Endothelial Cell Density (ECD)

In this study, the condition of corneal endothelial cells was analyzed in a group of patients with type 2 diabetes compared to a control group of patients without diabetes. A significantly lower ECD was observed in the group of patients with type 2 diabetes compared to the control group. The mean ECD value differed by 5.66% between the groups. It is important to emphasize that the lower ECD values in the diabetic group occurred despite good glycemic control.

The obtained results are similar to those reported by Choo et al., who compared corneal endothelial parameters in 100 patients with type 2 diabetes and a control group. Their study also found a statistically significant reduction in ECD in diabetic patients compared to the control group, with a mean ECD difference of 4.5% between groups. Additionally, they reported that ECD decreases as diabetic retinopathy progresses [4].

Tasli et al. conducted a study with 195 patients with type 2 diabetes, comparing their data with 100 patients in a control group. They also found lower ECD values in diabetic patients. The change in ECD observed in the present study is consistent with the findings of Tasli et al. regarding the same parameter. Furthermore, Tasli et al. highlighted the presence of a negative correlation between ECD and diabetes duration, HbA1c levels, and the urine albumin-to-creatinine ratio (ACR) in diabetic patients [8].

Another study examining a similar research question was conducted by El-Agamy et al., who reported a significantly lower ECD (by 5.24%) in the diabetic group compared to the control group. The results obtained by El-Agamy et al. are consistent with the findings of the present study [15].

A meta-analysis conducted by Sudhir et al. included 1191 patients with type 2 diabetes and 121 individuals in a control group. The study analyzed ECD values in patients with varying levels of glycemic control. A significantly lower ECD was observed in the diabetic group compared to the control group. The findings on ECD values in the present study align with the results reported by Sudhir et al. [17].

Another study that yielded consistent results with the present research was conducted by Islam et al. They reported a significantly lower ECD in the diabetic group compared to controls. Additionally, they found even lower ECD values and more pronounced changes in endothelial morphology in patients with diabetes lasting more than 10 years, compared to those with a shorter disease duration [18].

However, Storr-Palausen et al. obtained results that differed from the previously mentioned studies and the current findings. They did not observe a statistically significant difference in ECD values between patients with well-controlled type 2 diabetes and the control group. However, they did identify a relationship between higher HbA1c levels (indicative of poorer diabetes control) and lower ECD values [19].

Another study with results differing from those in this analysis was conducted by Siribunkum et al., who investigated corneal endothelial parameters in diabetic and control groups. This was the only study in which higher ECD values were observed in diabetic patients. A limitation of this Thai study was the small sample size (60 eyes from 30 patients). Additionally, the potential influence of ethnic differences on the results cannot be ignored [20].

Lee et al. reported that endothelial cell morphology differs significantly between diabetic and non-diabetic groups. Changes in ECD and endothelial morphology were more pronounced in patients with diabetes lasting over 10 years compared to those with a shorter disease duration. However, it is important to note that their study did not take into account glycemic control levels in diabetic patients [21].

Finally, Inoue et al. observed a significant ECD reduction of 4.1% in patients with type 2 diabetes compared to the control group. After analyzing data from 1394 patients, they concluded that age—not diabetes—was the only variable influencing endothelial cell density and morphology [22].

### 4.4. Coefficient of Variation in Endothelial Cell Size (CV)

CV expresses the variability in endothelial cell size, which, under physiological conditions, is relatively uniform. In this study, no significant difference was observed in CV values between the group of patients with well-controlled type 2 diabetes and the control group.

Sudhir et al., based on data from a large patient group, did not find a statistically significant difference in CV values between diabetic and control patients. Similar findings regarding CV values were also reported by Islam et al. [18].

A study led by Storr-Palausen yielded consistent results, showing no statistically significant difference in CV between diabetic patients with good glycemic control and controls [19].

However, Choo et al. found a statistically significant increase in CV values, indicating greater endothelial cell variability (polymegethism) in the diabetic group [4]. This result contradicts the current study findings, as well as the previously mentioned studies. The lack of differences in CV, similar to %HEX values, in the current study may be attributed to good diabetes control in the study population, whereas Choo et al. analyzed a more diverse diabetic group.

Tasli et al. also observed an increase in CV values in diabetic patients compared to the control group. However, the results of the current study differ from those obtained by Tasli et al. A key difference between the two studies is that Tasli et al. included both well-controlled and poorly controlled diabetic patients, whereas the present study only included patients with good diabetes control [8].

Lee et al. reported higher CV values in diabetic patients than in controls and emphasized that CV values were correlated with diabetes duration [21].

El-Agamy et al. observed a significant increase in CV values in diabetic patients compared to controls [15].

Siribunkum et al. also described increased polymegethism (higher CV values) in the diabetic group compared to controls; however, the differences they reported were not statistically significant [20].

### 4.5. Percentage of Hexagonal Cells (%HEX)

The percentage of hexagonal cells describes the stability of cell shape, which under physiological conditions has a characteristic, regular, hexagonal structure. A decrease in the %HEX parameter indicates an increase in pleomorphism.

In the present study, %HEX did not differ significantly between the groups. In the group of patients with type 2 diabetes, it was slightly lower than in the control group. However, this difference was not statistically significant.

El-Agamy et al. reported that the %HEX value did not differ significantly between the study and control groups. The results obtained by El-Agamy et al. are consistent with those in the present study [15].

Similar results were presented in a meta-analysis by Sudhir et al., who reported no significant differences in the %HEX parameter between the group of patients with type 2 diabetes and the control group [17].

Another study with findings consistent with those of the present study was conducted by Islam et al. The %HEX parameter did not differ significantly between the compared groups [18].

Storr-Palausen et al. obtained results that were consistent with those in this study. No statistically significant difference was observed in the %HEX parameter between the group of patients with type 2 diabetes with good glycemic control and the control group [19].

Choo et al. obtained different data regarding the percentage of hexagonal cells. In the group of diabetic patients, they observed a lower %HEX compared to non-diabetic patients. This discrepancy in results may be due to the fact that the researchers studied a group of patients with type 2 diabetes with varying levels of disease control. Meanwhile, the data obtained in the present study comes from a group of patients with well-controlled type 2 diabetes, where HbA1c levels remained below 7%. It can be inferred that poor glycemic control may lead to increased pleomorphism within the corneal endothelium. Choo et al. also noted that %HEX decreases with the progression of diabetic retinopathy [4].

Tasli et al. obtained different results, noting lower %HEX values in the group of patients with type 2 diabetes compared to the control group. However, it should be mentioned that their analysis included data collected from patients with type 2 diabetes without verifying the control of the underlying disease [8].

Siribunkum et al. reported an increase in pleomorphism in the diabetic group, but the decrease in %HEX they reported was not statistically significant [20].

### 4.6. Central Corneal Thickness (CCT)

Central corneal thickness is a parameter that indirectly describes the efficiency of endothelial cells as a pump, maintaining the corneal stroma in a state of proper hydration. Endothelial dysfunction leads to the impairment of its function, which manifests as excessive aqueous humour influx into the stroma. This is observed as corneal edema, which, in advanced stages, leads to reduced corneal transparency and, consequently, decreased visual acuity.

In the present study, no statistically significant difference in corneal thickness was found between the group of patients with type 2 diabetes and the control group.

Kotecha et al. did not observe a significant difference in CCT between groups of patients with and without diabetes [23]. Meanwhile, Lee et al. stated that CCT is closely related to the duration of diabetes and is slightly greater in the group of diabetic patients than in the group of non-diabetic patients [21].

The findings reported by El-Agamy et al. align with the results of the current study [15]. No significant difference in the CCT parameter between groups was observed. Similar findings were reported by Sudhir et al. [17].

Tasli et al. reported that CCT values in the group of patients with type 2 diabetes were higher than in the control group. The study analyzed data from patients regardless of the level of glycemic control [8].

Storr-Palausen et al. obtained results that differed from those of the present study. They reported significantly higher CCT values in the study group compared to the control group [17]. Similar findings were published by Siribunkum et al., who found significantly higher CCT values in the group of diabetic patients compared to the control group [20].

Altay et al. presented results consistent with those obtained in the present study, indicating no significant differences in the CCT parameter when comparing patients with type 2 diabetes who had been living with the disease for more than 10 years versus those with a shorter disease duration [24].

In studies conducted by Lee et al. and Briggs et al., it was noted that changes in the corneal endothelium and increased CCT become more pronounced as diabetes duration increases and are more evident in cases where diabetes has lasted for more than 10 years [21,25].

In summary, when comparing the results of the present study concerning endothelial cell status in the diabetic group to the literature, it can be inferred that long diabetes duration and poor glycemic control may lead to endothelial dysfunction. In this study, data were analyzed from patients with type 2 diabetes lasting between 1 and 10 years with good glycemic control, which may explain the lack of significant differences in CV, %HEX, and CCT in the diabetic group compared to the control group.

The novelty of our study lies in its exclusive focus on patients with well-controlled type 2 diabetes mellitus, in contrast to many previous studies that included heterogeneous diabetic populations. By selecting only patients with HbA1c ≤ 7% and applying strict exclusion criteria to minimize confounding factors, we were able to analyze subtle endothelial changes in a metabolically stable cohort. This targeted approach enhances the internal validity of our findings and provides new insights into early corneal endothelial alterations associated with well-controlled type 2 diabetes mellitus. Further studies involving larger patient cohorts and accounting for variables such as age, sex, and hormonal changes related to menopause are warranted to better understand the multifactorial influences on corneal endothelial health in diabetic populations.

## 5. Conclusions

ECD is lower in the group of patients with well-controlled type 2 diabetes compared to non-diabetic patients. Parameters describing the morphology of corneal endothelial cells, including %HEX and CV, do not differ significantly between the groups. CCT in patients with well-controlled type 2 diabetes is comparable to CCT values in non-diabetic patients. Despite the lower ECD in diabetic patients, the functional status of the cornea, as expressed by visual acuity and central corneal thickness, remains unchanged. Although type 2 diabetes negatively affects endothelial cell density, the lack of significant differences in their morphology and corneal thickness suggests that good glycemic control may reduce the negative impact of diabetes on the corneal endothelium.

## Figures and Tables

**Table 1:** Inclusion and Exclusion Criteria for Patient Recruitment.

Inclusion Criteria	Exclusion Criteria
-Informed consent to participate in the clinical study	-History of previous ophthalmic surgeries
-Age between 60 and 80 years	-Active ocular inflammatory condition
-Diagnosis of type 2 diabetes treated with oral medications for 1 to 10 years (for the study group)	-History of uveitis
-Well-controlled diabetes (HbA1c ≤ 7%)	-Use of contact lenses
	-Corneal scars, dystrophies
	-History of ocular trauma
	-Post-traumatic cataract, congenital cataract
	-Documented episodes of elevated IOP
	-Glaucoma
	-Pseudoexfoliation syndrome
	-Lens subluxation
	-ECD < 1500 cells/mm^2^
	-Difficulties in visualizing endothelial cells
	-Axial hyperopia (Axl < 21.0 mm)
	-Axial myopia (Axl > 26 mm)
	-Autoimmune diseases other than diabetes

**Table 2:** The characteristics of the group of patients with type 2 diabetes (DM+).

Variable	*n* (%)/Mean ± SD	Median (Q1; Q3)	Range (Min–Max)
N	80 (100.0)	-	-
Gender	-	-	-
Female	43 (53.8)	-	-
Male	37 (46.2)	-	-
Age (years)	72.04 ± 5.60	73.00 (67.00; 76.25)	60.00 to 80.00
HbA1c (%)	6.77 ± 0.13	6.80 (6.70; 6.90)	6.50 to 7.00
Corneal Endothelial Parameters	-	-	-
ECD (cells/mm^2^)	2480.76 ± 303.48	2497.50 (2350.00; 2706.00)	1780.00 to 3011.00
CV	30.00 ± 4.81	29.00 (27.00; 32.00)	22.00 to 45.00
%HEX (%)	68.21 ± 4.93	68.00 (65.75; 71.00)	58.00 to 80.00
Other Parameters	-	-	-
CCT (μm)	542.22 ± 27.94	539.00 (523.00; 559.00)	461.00 to 612.00
BCVA	0.44 ± 0.18	0.40 (0.30; 0.60)	0.10 to 1.00
IOP (mmHg)	15.59 ± 2.84	15.00 (14.00; 18.00)	9.00 to 22.00

**Note:** SD—standard deviation; Q1—first quartile; Q3—third quartile.

**Table 3:** The characteristics of the group of patients without type 2 diabetes (DM−).

Variable	*n* (%)/Mean ± SD	Median (Q1; Q3)	Range (Min–Max)
N	80 (100.0)	-	-
Gender	-	-	-
Female	42 (52.5)	-	-
Male	38 (47.5)	-	-
Age (years)	73.22 ± 4.90	74.00 (70.00; 77.00)	60.00 to 80.00
Blood Glucose (mg/dL)	92.90 ± 4.08	92.00 (90.00; 95.25)	79.00 to 100.00
Corneal Endothelial Parameters	-	-	-
ECD (cells/mm^2^)	2629.64 ± 304.73	2635.50 (2414.25; 2781.75)	1977.00 to 3679.00
CV	29.12 ± 4.14	29.00 (26.00; 32.00)	21.00 to 43.00
%HEX (%)	68.86 ± 4.59	69.00 (66.00; 72.00)	54.00 to 79.00
Other Parameters	-	-	-
CCT (μm)	548.20 ± 24.84	547.00 (532.75; 566.25)	495.00 to 606.00
BCVA	0.50 ± 0.19	0.50 (0.40; 0.60)	0.10 to 1.00
IOP (mmHg)	15.38 ± 3.07	15.00 (13.00; 17.00)	9.00 to 25.00

**Note:** SD—standard deviation; Q1—first quartile; Q3—third quartile.

**Table 4:** Comparison of patients with type 2 diabetes (DM+) and without type 2 diabetes (DM−).

Variable	DM+	DM−	MD (95% CI)	*p*
Gender	-	-	-	>0.999 ^3^
Female	43 (53.8)	42 (52.5)	-	
Male	37 (46.2)	38 (47.5)	-	
Age (years)	72.04 ± 5.60	73.22 ± 4.90	−1.19 (−2.83; 0.46)	0.155
Corneal Endothelial Parameters	-	-	-	-
ECD (cells/mm^2^)	2480.76 ± 303.48	2629.64 ± 304.73	−148.88 (−243.84; −53.91)	0.002
CV	29.00 (27.00; 32.00)	29.00 (26.00; 32.00)	0.00 (−1.00; 2.00) ^1^	0.407 ^2^
%HEX (%)	68.21 ± 4.93	68.86 ± 4.59	−0.65 (−2.14; 0.84)	0.390
Other Parameters	-	-	-	-
CCT (μm)	542.22 ± 27.94	548.20 ± 24.84	−5.98 (−14.23; 2.28)	0.155
BCVA	0.44 ± 0.18	0.50 ± 0.19	−0.06 (−0.11; 0.00)	0.049
IOP (mmHg)	15.59 ± 2.84	15.38 ± 3.07	0.21 (−0.71; 1.14)	0.650

**Note:** Data are presented as the number of observations (% of group) for categorical variables, mean ± standard deviation for numerical variables with a normal-like distribution, or median (first quartile; third quartile) for numerical variables with a non-normal distribution. MD—mean or median difference ^1^ (DM+ vs. DM−). CI—confidence interval. Groups were compared using Student’s *t*-test for independent samples, the Mann–Whitney U test ^2^, or Pearson’s chi-square test ^3^.

## Data Availability

The data that support the findings of this study are available from the corresponding author upon reasonable request.
